# The complete genome sequence of *Cronobacter sakazakii* ATCC 29544^T^, a food-borne pathogen, isolated from a child’s throat

**DOI:** 10.1186/s13099-016-0150-0

**Published:** 2017-01-04

**Authors:** Seongok Kim, You-Tae Kim, Hyunjin Yoon, Ju-Hoon Lee, Sangryeol Ryu

**Affiliations:** 1 Department of Food and Animal Biotechnology, Department of Agricultural Biotechnology, Research Institute for Agriculture and Life Sciences, and Center for Food and Bioconvergence, Seoul National University, 1 Gwanak-ro, Gwanak-gu, Seoul, 08826 Republic of Korea; 2 Department of Applied Chemistry and Biological Engineering, Ajou University, 206 Worldcup-ro, Yeongtong-gu, Suwon, 16499 Republic of Korea; 3 Department of Food Science and Biotechnology and Institute of Life Science and Resources, Kyung Hee University, 1732 Deogyeong-daero, Giheung-gu, Yongin-si, Gyeonggi-do, 17104 Republic of Korea

**Keywords:** *Cronobacter sakazakii*, Complete genome sequence, Pathogenesis, Infant milk formula, Virulence factor

## Abstract

**Background:**

*Cronobacter sakazakii* is an emerging opportunistic pathogen that is associated with rare but life-threatening cases of severe diseases: meningitis, necrotizing enterocolitis, and sepsis in premature and full-term infants. However, the pathogenesis mechanism of this pathogen remains largely unknown. To determine its pathogenesis at the genomic level, the genome of *C. sakazakii* ATCC 29544^T^ was completely sequenced and analyzed.

**Results:**

The genomic DNA, containing a circular chromosome and three plasmids, is composed of 4,511,265 bp with a GC content of 56.71%, containing 4380 predicted open reading frames (ORFs), 22 rRNA genes, and 83 tRNA genes. The plasmids, designated pCSK29544_p1, pCSK29544_p2, and pCSK29544_p3, were 93,905-bp, 4938-bp, and 53,457-bp with GC contents of 57.02, 54.88, and 50.07%, respectively. They were also predicted to have 72, 6, and 57 ORFs without RNA genes.

**Conclusions:**

The strain ATCC 29544^T^ genome has *ompA* and *ibeB*-homologous *cusC* genes, probably associated with the invasion of human brain microvascular endothelial cells (BMECs). In addition, gene clusters for siderophore production (*iucABCD*/*iutA*) and the related transport system (*eitCBAD*) were detected in pCSK29544_p1 plasmid, indicating better iron uptake ability for survival. Furthermore, to survive under extremely dry condition like milk powder, this genome has gene clusters for biosynthesis of capsular proteins (CSK29544_00281-00284) and cellulose (CSK29544_01124-01127) for biofilm formation and a gene cluster for utilization of sialic acid in the milk (*nanKTAR*). The genome information of *C. sakazakii* ATCC 29544^T^ would provide further understanding of its pathogenesis at the molecular level for the regulation of pathogenicity and the development of a rapid detection method using biomarkers.

**Electronic supplementary material:**

The online version of this article (doi:10.1186/s13099-016-0150-0) contains supplementary material, which is available to authorized users.

## Background

*Enterobacter sakazakii* has been reclassified into seven species in the genus *Cronobacter* according to biochemical and genetic evaluations [1, 2]. Among them, *Cronobacter sakazakii* is a well-known opportunistic food-borne pathogen causing bacteremia, meningitis and necrotizing enterocolitis, particularly in low-birth-weight neonatal infants. This species is a Gram-negative, rod-shaped, peritrichous and yellow-pigmented facultative anaerobe belonging to the *Enterobacteriaceae* family [3]. *C. sakazakii* has been often found in human and infant gut microbiota [4, 5]. Although *C. sakazakii* food-borne outbreaks are quite low, the fatality to infants generally ranges from 40 to 80% [6]. Interestingly, *C. sakazakii* was reported to produce capsular or biofilm materials for its own protection from extremely dry conditions, as in formula milk powder, substantiating the high survival ability of *C. sakazakii* in the milk powder [7]. After human infection, *C. sakazakii* can invade the intestinal epithelial cells and even the brain microvascular endothelial cells (BMECs), demonstrating its potentials to cause meningitis [8]. Therefore, the biocontrol and regulation of *C. sakazakii* are urgently required. However, *C. sakazakii* is resistant to some antibiotics, indicating a problem with antibiotic therapies against *C. sakazakii* [9, 10], and its pathogenicity mechanism remains unknown. Recently, to unveil the knowledge about the *Cronobacter* ecology, multilocus sequence typing (MLST) analysis using seven housekeeping genes has been established to identify the diversity of the *Cronobacter* genus from various sources, and its application has facilitated understanding of the evolutionary relationships and environmental fitness of *Cronobacter* species [2]. In this study, to understand its infection and pathogenesis at the molecular level, the genome of a representative *C. sakazakii* type strain, ATCC 29544^T^, was completely sequenced and analyzed in this study using bioinformatics. This genome information would provide the researchers with genomic insights into the virulence and pathogenicity mechanisms of this species for the further development of a rapid detection method and a novel biocontrol strategy.

## Methods

### Growth conditions and DNA isolation

*Cronobacter sakazakii* ATCC 29544^T^ was routinely cultivated using Luria-Bertani (LB) medium at 37 °C with shaking at 220 rpm. Bacterial cells were harvested in the mid-exponential growth phase using centrifugation at 16,000×*g* for 1 min and its genomic DNA was isolated using G-spin™ Genomic DNA Extraction Kit for Bacteria (iNtRON Biotechnology, Seongnam, South Korea). The concentration and purity of extracted DNA were determined by NanoVue (GE healthcare, Little Chalfont, United Kingdom).

### Genome sequencing and assembly

The complete genome of *C. sakazakii* ATCC 29544^T^ was sequenced at Macrogen, Seoul, South Korea, using a hybrid of PacBio RS II (Pacific Biosciences, Menlo Park, CA, USA) and Illumina HiSeq 2500 (San Diego, CA, USA). The sequence reads from PacBio RS II and Illumina HiSeq 2500 platforms were assembled using HGAP (version 2.0) and ALLPATHS-LG (version r47449), respectively. The final genome coverages were on average 1321 X Illumina and 73 X PacBio, respectively.

### Genome annotation

The ORFs were predicted using Glimmer3 [11] and GeneMark.hmm [12]. The gene prediction results were confirmed by manual curation. The genes of rRNA and tRNA were predicted using RNAmmer 1.2 [13] and tRNAscan-SE [14], respectively. The genome annotation was conducted using NCBI BLASTP [15] and a predicted protein analysis using InterProScan 5 [16] for the prediction of protein functions.

### The analysis of comparative genomes and phage-associated regions

Phage-associated gene clusters in the genome sequences of *C. sakazakii* ATCC 29544 were searched using PHAST server [17].

### Quality assurance

*Cronobacter sakazakii* ATCC 29544^T^ was obtained from American Type Culture Collection (ATCC) and its morphological observation using a transmission electron microscopy (TEM) showed a traditional shape of *C. sakazakii* as a short rod (Additional file 1: Figure S1). In addition, this strain was confirmed to *C. sakazakii* using 16S rRNA gene sequencing. For genome sequencing, the raw read sequences were selected and assembled when their quality scores were more than 40 as cutoffs. The complete genome sequence after genome assembly was also used for confirmation using ANI analysis with previously reported complete genome sequences of *C. sakazakii*.

## Initial findings

### General genome properties

**Fig. 1:**
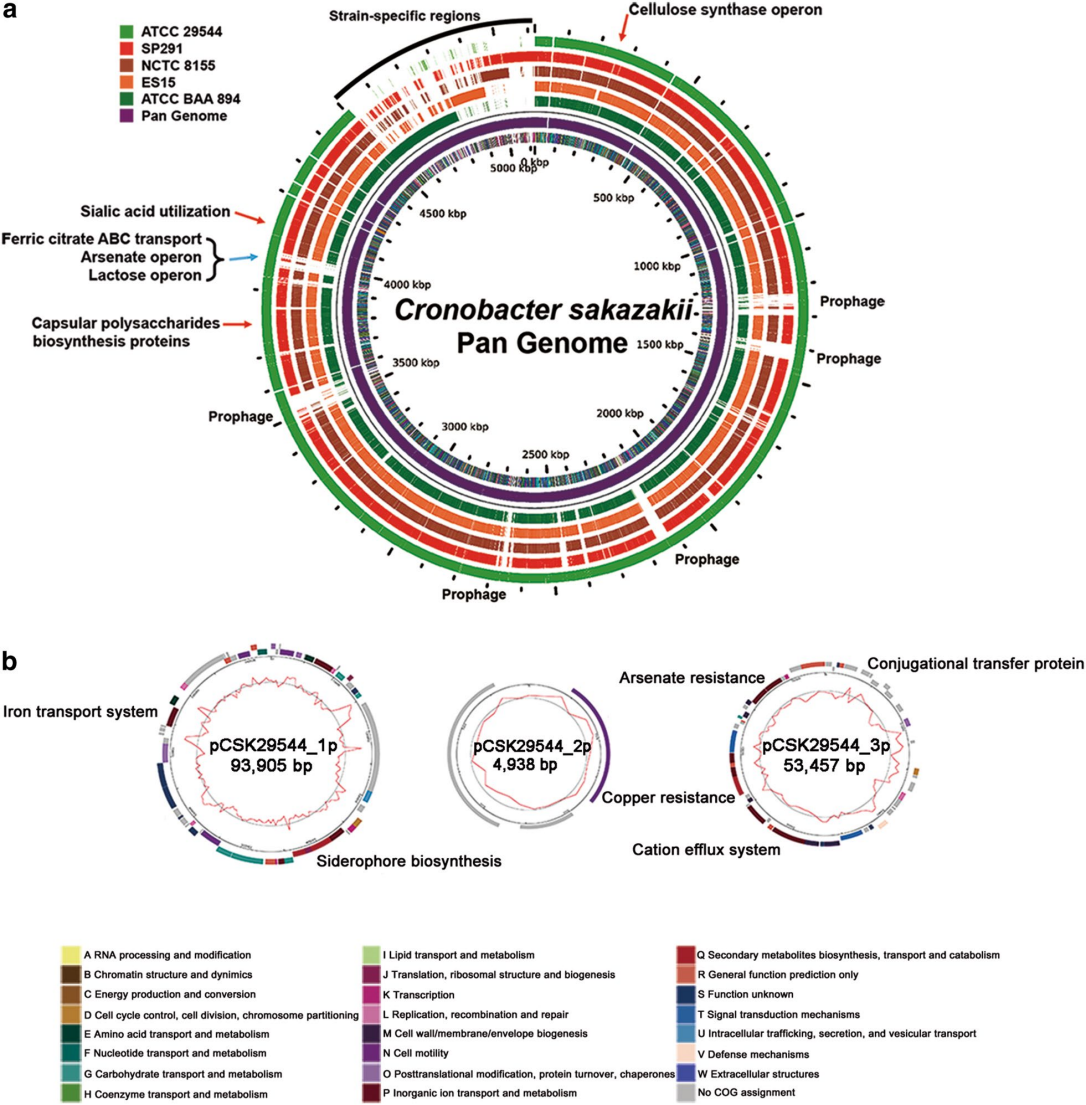
**a** Pan genome map of five *C. sakazakii* strains using GView [34]. The cut off value of BLASTN homology was 80%. The *inner circle* represents COG functional categories of *C. sakazakii* ATCC 29544^T^. *Red arrows* indicate gene cluster conserved in all *C. sakazakii* strains and *blue arrows* indicate ATCC 29544^T^—specific gene clusters. *Black curved bar* indicates other *C. sakazakii* strain-specific regions. **b** Plasmid maps from *C. sakazakii* ATCC 29544^T^. The *curved bars* in the *outer circle* indicate the predicted ORFs by strand, and colored by COG categories. The *inner circle with red* peaks indicates the G + C content

The complete genome of *C. sakazakii* ATCC 29544^T^ is composed of a circular chromosome and three plasmids (Fig. 1). The chromosome is 4,511,265 bp in DNA length with a GC content of 56.71%, 4380 ORFs, 22 rRNA genes, and 83 tRNA genes. The plasmids, designated pCSK29544_p1, pCSK29544_p2 and pCSK29544_p3, are 93,905, 4938 and 53,457 bp in DNA length with GC contents of 57.02, 54.88 and 50.07%, respectively. The plasmids have predicted ORFs of 72, 6, and 57.

### Pathogenesis and virulence factors

*Cronobacter sakazakii* infects human neonates and infants via mostly contaminated reconstituted infant formula milk, causing serious human diseases, including bacteremia, necrotizing enterocolitis, and even meningitis [6]. The high survival rate of *C. sakazakii,* even under extremely dried conditions, as in milk formula powder, has not been investigated at the molecular level. To understand this extraordinary property of *C. sakazakii*, molecular studies have been recently performed. Interestingly, the gene clusters associated with biosynthesis of capsular polysaccharides (CSK29544_00281-00284) and cellulose (CSK29544_01124-01127) were detected. Recently, the biofilm formation and cellulose (as a component of biofilm) production of *C. sakazakii* were experimentally confirmed [18, 19], suggesting that these gene clusters may be involved in the biofilm formation. This biofilm formation of *C. sakazakii* may contribute to the survival in the infant formula conditions [19]. However, exopolysaccharide (EPS) production was not observed in *C. sakazakii* ATCC 29544^T^ [20], suggesting that capsular polysaccharide gene cluster may be associated with biofilm formation, not with EPS production. In particular, *C. sakazakii* is the only *Cronobacter* species that has the *nanKTAR* gene cluster to utilize sialic acid [21]. Interestingly, *C. sakazakii* ATCC 29544^T^ has this gene cluster (CSK29544_00587-590), indicating its ability to use it. This unique ability may be involved in its adaptation to the milk conditions because sialic acid is one of the components of milk [22].

Upon human infection, *C. sakazakii* invades brain microvascular endothelial cells (BMECs), barriers to protect the brain from infection of meningitic pathogens, including *Escherichia coli* K1 and *Neisseria meningitides*, which cause meningitis in neonates and infants [23, 24]. Outer membrane protein A (OmpA) of *C. sakazakii* is a critical determinant, contributing in vitro invasion of human BMECs by enhancing cell adhesion [24]. In addition, a few genes (*ibeA, ibeB,* and *yijP*) in meningitic *E. coli* were also suggested to be associated with the invasion of human BMECs [25–27]. Interestingly, two genes, *ompA* (CSK29544_03699) for BMEC adhesion and the *ibeB*-homologous *cusC* (pCSK29544_3p0028) for the penetration of BMECs, were detected in the genome of *C. sakazakii* ATCC 29544^T−^, suggesting this strain may invade human BMECs. However, *ibeA* and *yijP* were not detected in the genome. It is noteworthy that this *ibeB*-homologous *cusC* is located in the gene cluster of the complete copper- and silver-resistance cation efflux system as encoded by *cusABCF* (CSK29544_3p0026–CSK29544_3p0031) in the plasmid pCSK29544_3p, suggesting this BMEC invasion ability may be transferrable to other *C. sakazakii* or that the strain ATCC 29544^T^ may have been acquired from other *C. sakazakii* for human BMEC invasion. The presence of a conjugational transfer protein encoded by *traX* (CSK29544_3p0007) supports this hypothesis.

*Cronobacter sakazakii* ATCC 29544^T^ harbors three plasmids, pCSK29544_1p (1p), pCSK29544_2p (2p), and pCSK29544_3p (3p). Interestingly, two large plasmids (1p and 3p) encode virulence-associated genes, related to iron uptake and BMEC invasion. Therefore, these two plasmids may contribute to host dominance and pathogenesis, respectively.

Iron is an essential element for dominant survival and colonization via bacterial competition for iron uptake because it plays an important role in the electron transport system for energy production [28]. To accomplish this dominant survival and colonization against other bacteria, *C. sakazakii* has an iron acquisition system, including siderophore production (*iucABCD*/*iutA*) and an ABC-type transport system (*eitCBAD*) in the plasmid pESA3 [29]. The strain ATCC 29544^T^ also has this privileged iron acquisition system, including siderophore biosynthesis (*iucABCD*/*iutA*; CSK29544_1p0024–CSK29544_1p0028) and an ABC-type iron transport system (*eitCBAD*; CSK29544_1p0056–CSK29544_1p0059). However, this iron acquisition system is present in the plasmid 1p, similar to pESA3 of *C. sakazakii* BAA-894 [29]. Interestingly, *C. sakazakii* BAA-894 has multiple copies of pESA3 plasmids in a host, which is highly homologous to the plasmid 1p, suggesting the plasmid 1p may be multi copied, too [30]. These results indicate that the host strain may take advantage of better iron uptake ability in the given environments, probably due to the presence of multiple copies of this iron acquisition system encoded in the plasmid 1p.

Recent studies have revealed that milk formula contains an at least six-times-higher arsenic concentration than that of breast milk, suggesting bacterial survival in milk formula may require arsenic resistance [31]. In addition to the previously mentioned BMEC invasion ability of *C. sakazakii* ATCC 29544^T^ via the *ibeB*-homologous *cusC* gene in the cation efflux system, the plasmid 3p has an arsenic resistance system as encoded by *arsRDABC*, suggesting the strain ATCC 29544^T^ may have arsenic resistance activity for survival in formula milk powder and that this ability may have been acquired via plasmid conjugational transfer.

### Comparative genome analysis

**Fig. 2:**
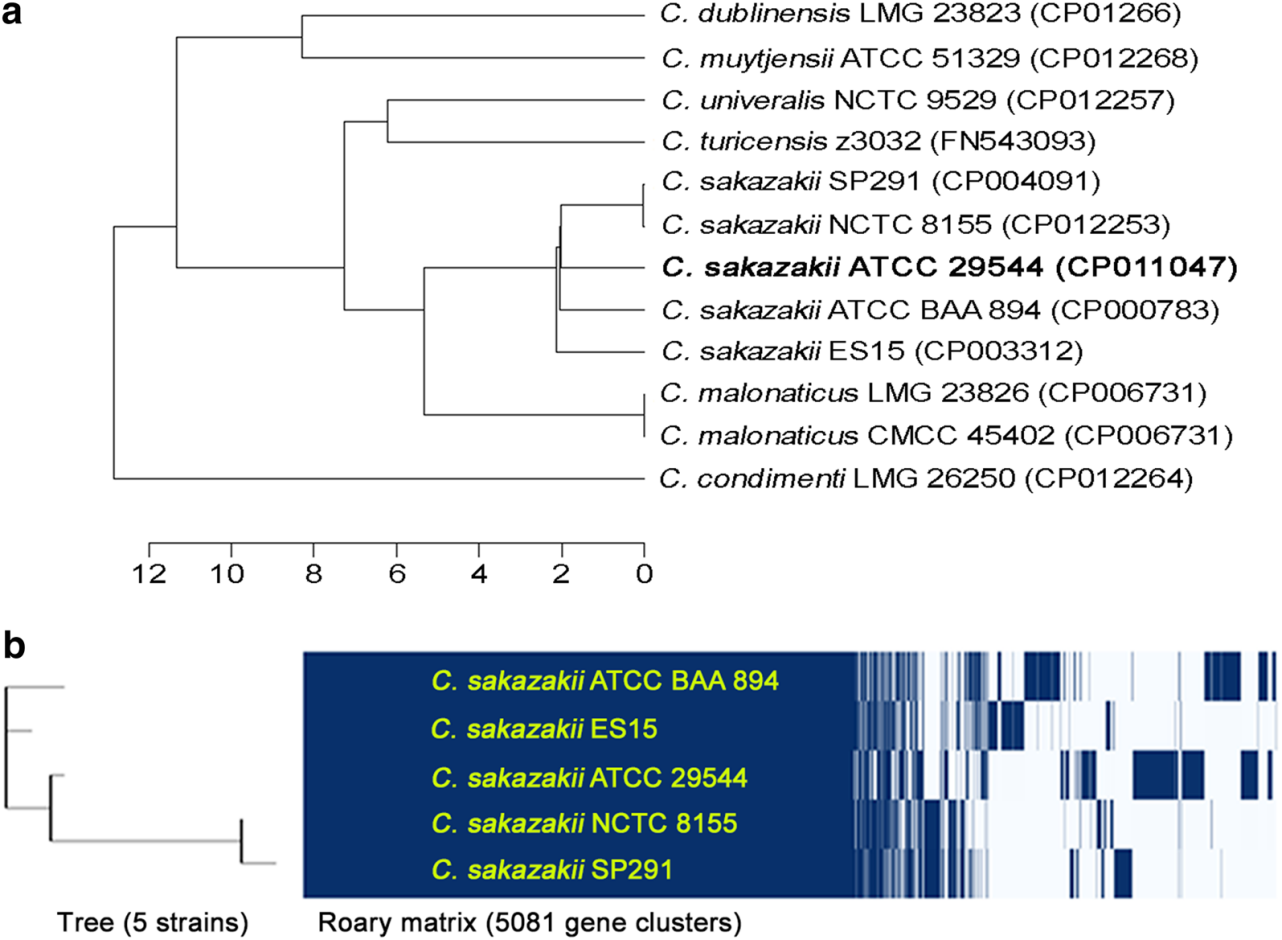
**a** Phylogenetic tree analysis of *C. sakazakii* ATCC 29544^T^ and other *Cronobacter* species strains by average nucleotide identity (ANI) using JSpecies. **b** Roary matrix-based protein sequence comparison and the associated taxonomical tree of five *C. sakazakii* strains [35]

To date, five complete genome sequences of *C. sakazakii* and their annotation data are available in the GenBank database. To compare these genomes, whole genomic DNA sequence-based average nucleotide index (ANI) analysis and Roary matrix-based protein sequence analysis were performed. ANI analysis showed that *C. sakazakii* NCTC 8155 and SP291 are the most closely related, forming a taxonomical group, and the strains ATCC 29544^T^, ATCC BAA894, and ES15 are also similar to this group (Fig. 2a). Subsequent Roary matrix-based protein sequence analysis of these five genomes supports this relationship among them (Fig. 2b). However, the strain ATCC 29544^T^ is more closely related to the group in the Roary matrix-based tree.

Moreover, pan genome analysis of these five genomes was conducted and their strain-specific regions were detected (Fig. 1). Interestingly, three functional gene clusters and seven prophage regions are ATCC 29544^T^-specific. In addition to the iron acquisition system consisting of siderophore production and ABC-type transport system in the plasmid 1p, ATCC 29544^T^-specific iron uptake-associated gene cluster containing *fecABDCEI* for dominant uptake of ferric dicitrate is present in the chromosome, probably related to survival in the iron-limited condition [32]. The second arsenic resistance gene cluster consisting of *arsRCB* for arsenate sensing, reduction, and efflux pumping was also detected, which is similar to the gene cluster in the plasmid 3p, probably for dominant survival in the human gut containing low concentration of arsenic after human infection [33] (Additional file 1: Fig. S2A). Comparative analysis of their protein sequences showed that they are >94% identical to each other, probably for synergistic arsenic resistance activity. Unlike other two gene clusters shared by the chromosome and plasmid, two copies of lactose operons are present in the chromosomes of ATCC 29544^T^, NCTC 8155, and SP291, probably related to the Roary matrix-based tree (Fig. 2b). While one of these operons (CSK29544_04271–04273) are shared by all *C. sakazakii* genomes, the second lactose operon (CSK29544_00439–00442) is ATCC 29544^T^-specific. This second lactose operon is similar to other lactose operon in *Klebsiella* and *Enterobacter* (data not shown), suggesting that this operon may be originated from other genus bacterium. The presence of multiple mobile elements containing transposases and relatively low protein sequence identity support this hypothesis (Additional file 1: Fig. S2B). Furthermore, pan genome analysis showed strain-specific region (Fig. 1). Most of genes in this region belong to prophages and they have very low homology among them, indicating that these prophages are also strain-specific. Interestingly, the strain ATCC 29544^T^ contains seven strain-specific prophage regions with five intact prophages and two incomplete prophages (Table 1). However, most of genes in the prophages encode hypothetical proteins.

**Table 1:** Genome statistics

Attribute	Value	% of Total^a^
Genome size (bp)	4,663,565	100
DNA coding (bp)	4,116,350	88.27
DNA G + C (bp)	2,641,511	56.64
Total genes	4620	100
Protein coding genes	4515	97.72
RNA genes	105	2.27
rRNA operons	22	0.48
tRNA genes	83	1.8
Genes with function prediction	3212	69.52
Predicted prophages	7	6.18
CRISPR repeats	2	0.04

^a^The total is based on either the size of the genome (chromosome and 3 plasmids) in base pairs or the total number of total genes in the annotated genome

## Future directions

This genome information of *C. sakazakii* ATCC 29544^T^ provides genomic insights into the presence of various virulence factors and their associated pathogenesis mechanisms at molecular level. In addition, this genome information showed that the strain ATCC 29544^T^ has several distinct features probably contributing to dominant survival under the given habitats and even various stress conditions like extremely dry or nutrient-limited conditions. Therefore, this genome information would be useful for further development of a novel strategy for control of *C. sakazakii* and of rapid detection method using strain-specific DNA markers in genomic level.

## Additional file

**Additional file 1: Figure S1.** The morphology of *C. sakazakii* ATCC 29544 imaged by energy-filtering transmission electron microscopy (EF-TEM). EF-TEM photograph was obtained by 2% uranyl acetate on copper grids and examined with ET-TEM at a voltage of 120 kV (LIBRA 120, Zeiss, Oberkochen, Germany). **Figure S2:** Comparative analysis of two ATCC 29544-specific gene clusters: (A) *lac* operon and (B) arsenic resistance. The amino acid sequence identities between associated genes are indicated as percentages.

## Abbreviations

BMECs: brain microvascular endothelial cells
ANI: average nucleotide identity
LB: Luria–Bertani medium
MLST: multilocus sequence typing
TEM: transmission electron microscopy
COG: clusters of orthologous groups
