# Supplementary material for: The complete genome sequence of Cronobacter sakazakii ATCC 29544T, a food-borne pathogen, isolated from a child’s throat
Source: Gut Pathog. 2017 Jan 4;9:2. doi: 10.1186/s13099-016-0150-0 (PMC5209807; doi:10.1186/s13099-016-0150-0)
Supplement: Supplementary file 1 — Additional file 1: Figure S1. The morphology of C. sakazakii ATCC 29544 imaged by energy-filtering transmission electron microscopy (EF-TEM). EF-TEM photograph was obtained by 2% uranyl acetate on copper grids and examined with ET-TEM at a voltage of 120 kV (LIBRA 120, Zeiss, Oberkochen, Germany). Figure S2: Comparative analysis of two ATCC 29544-specific gene clusters: (A) lac operon and (B) arsenic resistance. The amino acid sequence identities between associated genes are indicated as percentages. [file 13099_2016_150_MOESM1_ESM.docx]

**Figure S1.**

The morphology of *C. sakazakii* ATCC 29544 imaged by energy-filtering transmission electron microscopy (EF-TEM). EF-TEM photograph was obtained by 2% uranyl acetate on copper grids and examined with ET-TEM at a voltage of 120 kV (LIBRA 120, Zeiss, Oberkochen, Germany).

**
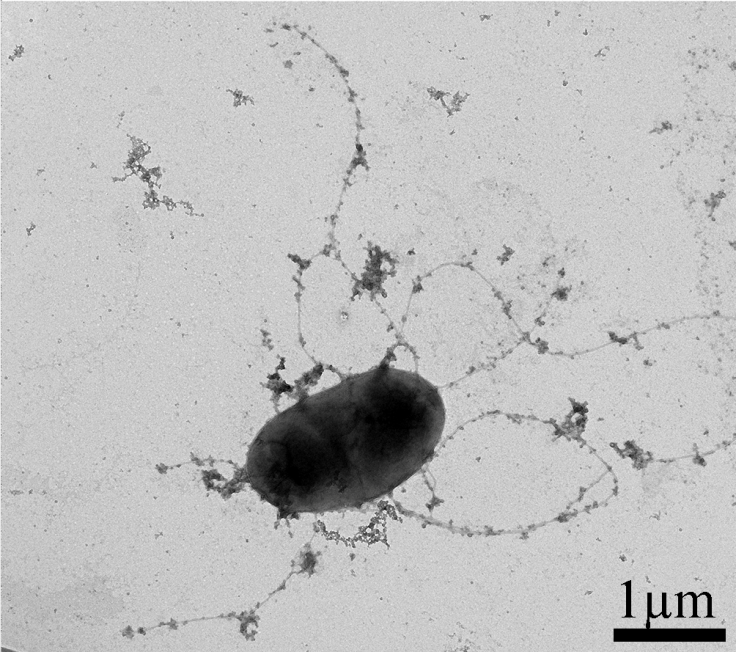
**

**Figure S2.**

Comparative analysis of two ATCC 29544-specific gene clusters: (A) *lac* operon and (B) arsenic resistance. The amino acid sequence identities between associated genes are indicated as percentages.
